# A pantothenic acid deficiency outbreak causing mortality and neuromuscular disorders in newborn and nursery piglets

**DOI:** 10.1186/s40813-026-00527-z

**Published:** 2026-06-17

**Authors:** Sara Isabel Loscertales, Anna Romagosa, Laura Martino, Marina Cid-Cañete, Sofía Bosco, Mariona Leiva-Forns, Joaquim Segalés

**Affiliations:** 1 Servei de Diagnòstic de Patologia Veterinària, Universitat Autònoma de Barcelona, Bellaterra, 08193 Spain; 2 Departament de Sanitat i Anatomia Animals, Universitat Autònoma de Barcelona (UAB), Bellaterra, 08193 Spain; 3 Pig Improvement Company (PIC), Pau Vila 22, Sant Cugat del Valles, 08174 Spain; 4 Unitat Mixta d’Investigació IRTA-UAB en Sanitat Animal, Centre de Recerca en Sanitat Animal (CReSA), Campus de la Universitat Autònoma de Barcelona (UAB), Bellaterra, 08193 Spain; 5 IRTA Programa de Sanitat Animal, Centre de Recerca en Sanitat Animal (CReSA), Campus de la Universitat Autònoma de Barcelona (UAB), Bellaterra, 08193 Spain; 6 WOAH Collaborating Center for Research and Control of Emerging and Re-Emerging Pig Diseases (IRTA-CReSA), Bellaterra, 08193 Spain

**Keywords:** Swine, Piglet, Vitamin B5, Pantothenic acid, Deficiency

## Abstract

**Background:**

The present case report describes the clinical and pathological outcomes of an outbreak of piglet mortality and neuromuscular disorders in newborn and nursery piglets caused by vitamin B5 deficiency.

**Case presentation:**

A farrow-to-wean 750 sow-farm in Spain experienced abortions and respiratory signs in sows related to swine influenza during the first week of February 2025. It was followed by an unusual high piglet mortality rate within the first week post-farrowing, reaching up to 65% and persisting for seven weeks. Piglets were born apparently healthy, and colostrum intake was considered normal. Nevertheless, on the second day of life, they started showing apathy, ataxia, and prostration, most of them usually dying spontaneously on the third day of life. At the same time, at least 14 recently weaned piglets exhibited similar clinical signs and subsequently died. However, sows were completely unaffected. Initial antibiotic treatments were ineffective. Seven piglets were subjected to a complete necropsy. Five showed the above-mentioned neurological signs and two were completely healthy, which served as non-affected control animals. No relevant gross lesions were found. Histopathologically, neuronal bodies from cervical, thoracic, and lumbar spinal cord had variable degrees of central chromatolysis, cytoplasmic vacuolation, and swelling, with displaced nuclei and occasional necrosis. Some of the sampled sciatic nerves had mild-to-intense axonal swelling and fragmentation. Microscopic lesions were absent in the two non-affected pigs. Pantothenic acid deficiency was presumptively diagnosed, and all newborn piglets were treated with a vitamin B complex. The condition did not re-appear after treatment.

**Conclusions:**

Insufficient pantothenic acid intake should be among the differential diagnoses of piglets with ataxia, muscle weakness and mortality. A fast diagnosis based on complete nervous system sampling is key to diagnose and counteract this condition.

**Supplementary Information:**

The online version contains supplementary material available at https://doi.org/10.1186/s40813-026-00527-z.

## Background

Pantothenic acid (PA), also known as vitamin B5, is a water-soluble nutrient, precursor of coenzyme A (CoA) biosynthesis. This coenzyme is responsible for carrying acyl groups during the Krebs cycle for energy-production [1, 2]. This is the reason why PA is considered critical in the metabolism and synthesis of carbohydrates, protein, and fat. Moreover, pantothenic acid, via CoA, is also involved in the synthesis of multiple neurotransmitters and steroid hormones [1, 2]. Although exceptionally rare in humans [3], PA deficiency in pigs has been both observed naturally on-farm as well as assessed experimentally [4–8]. Moreover, it has been described in many other animal species [2].

The classical clinical manifestation of PA deficiency in pigs is “goose-stepping” [6, 9], which evolves, in severe cases, into inability to stand or use their hind limbs [6, 9, 10]. Locomotor disturbances are associated with demyelination in the sciatic and brachial nerves, as well as in the dorsal root ganglia. Disruption of normal myelin function due to impaired turnover has also been documented [10]. Additional signs include impaired growth, anorexia, diarrhoea, ulceration of the gastrointestinal (GI) tract, dermatological lesions, and a sparse or thin hair coat [10]. In blood tests, normocytic anaemia and reduced immune responses have been described [11]. B5 hypovitaminosis in lactating pigs is also characterized by impaired suckling reflexes and reduced tongue mobility [10]. Death due to this deficiency has been also reported, reaching maximum values of 40% in lactating pigs [7].

Vitamin B5 is naturally present in whole grains, vegetables, eggs, milk, meat, and other nutrients used in the feedstuff [10]. In neonatal piglets, the requirements for PA are met primarily through colostrum and sow’s milk, supplemented as necessary through specialized diets formulated to support rapid growth and optimal health. A basal requirement of approximately 12.5 mg of PA per kilogram of dietary solids is considered essential for normal development [12]. However, higher dietary concentrations are recommended to enhance immune function and to prevent clinical manifestations such as severe diarrhoea and locomotor incoordination [13, 14]. Most of the reports assessing B5 hypovitaminosis in newborns or weaned piglets in the field come from low levels of PA inclusion in the diet of the sows or pre-stater diets [5–7].

Any neonatal piglet mortality cause is a significant concern in swine production, and the present report describes a case of PA deficiency responsible for high mortality and neuromuscular disorders in piglets. The clinical condition was resolved through vitamin B5 supplementation to newborn piglets and increasing the dietary dose in sows.

## Case presentation

### Farm description

The affected farm consisted of a high health 750 farrow-to-wean herd, with sows from Pietrain and Duroc genetic lines, and whose piglets were grown from six to thirty kilograms in the nursery. The herd had a high health status regarding common swine diseases (seronegative against porcine reproductive and respiratory syndrome virus [PRRSV], *Mycoplasma hyopneumoniae*, porcine epidemic diarrhea virus and transmissible gastroenteritis virus, and free from mange). Standard health management included vaccination against *Escherichia coli* and *Clostridium perfringens* (pre-farrowing) and *Erysipelothrix rhusiopathiae* and porcine parvovirus 1 (during lactation) in sows, and against porcine circovirus 2 (PCV2) and *Lawsonia intracellularis* in piglets at weaning.

### Clinical presentation

**Fig. 1:**
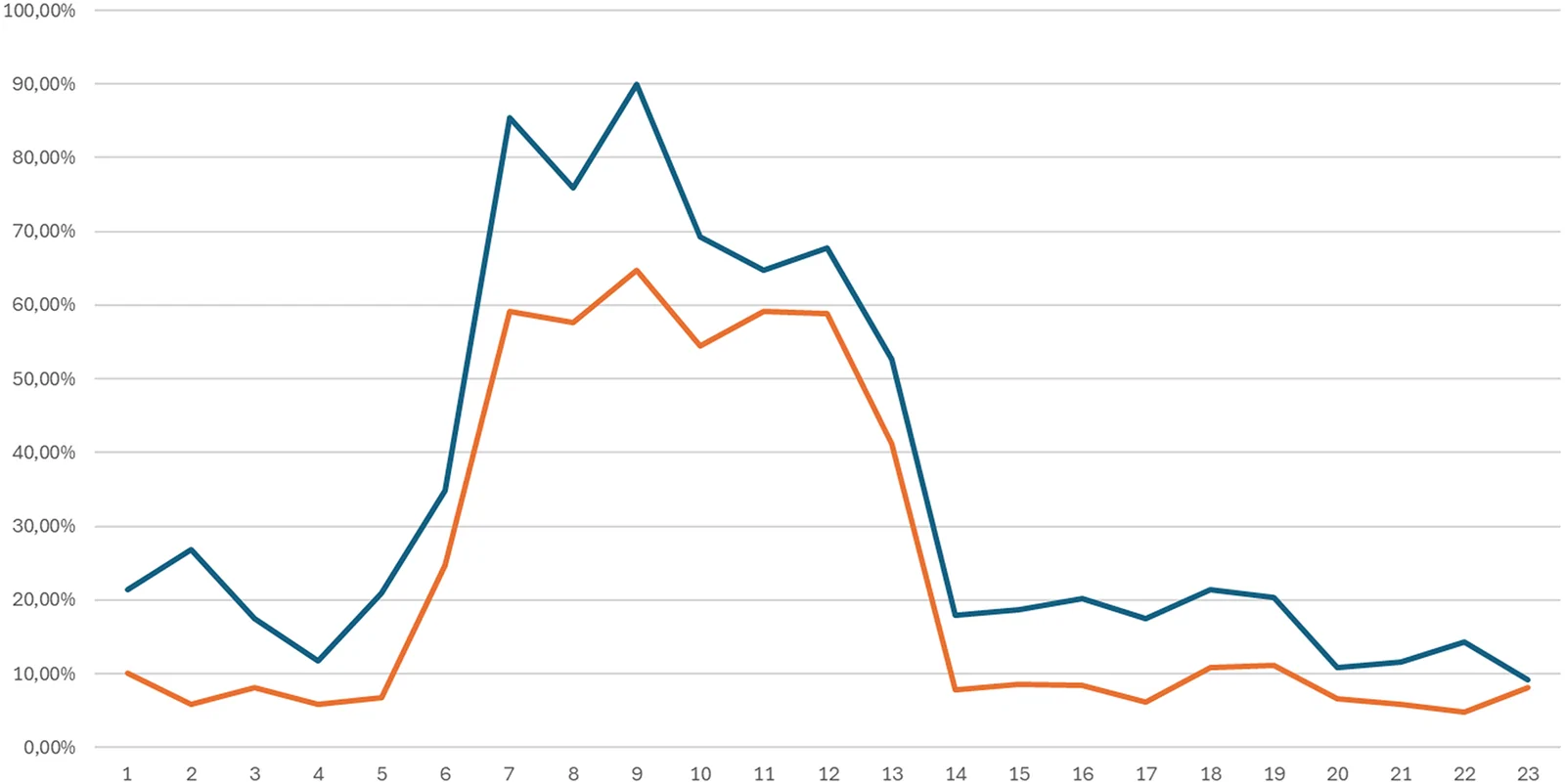
Percentage of piglet mortality over total born alive per week (X-axis: weeks of the year 2025). Blue line = total piglet mortality during lactation; Orange line = piglet mortality during the first week of life

In early February 2025, seven abortions and mild to moderate coughing were observed in sows. Flu caused by a pandemic H1N1 (pH1N1) influenza A virus was diagnosed by means of RT-PCR and subtyping, and the breeding herd was vaccinated with a commercial pH1N1 influenza vaccine. Two weeks later, high piglet mortality was experienced in the farrowing crates, with piglets appearing healthy at birth but dying around day three of life (Fig. 1). On-site necropsies revealed mesocolon, subcutaneous, and palpebral oedema in few animals, as well as scours in others. Based on such finding, samples from faeces and enteric tissues were analysed for digestive pathogen detection and PCR for *Clostridioides difficile* and RT-PCR for rotavirus A, which resulted positive. The Ct levels were 33.2 and 26.4, respectively. It was then decided to treat sows before farrowing parentally with 2 mg/ kg of body weight of marbofloxacin. Also, newborns were administered a dose of 2.5 mg/ kg of body weight tulathromycin at birth.

**Fig. 2:**
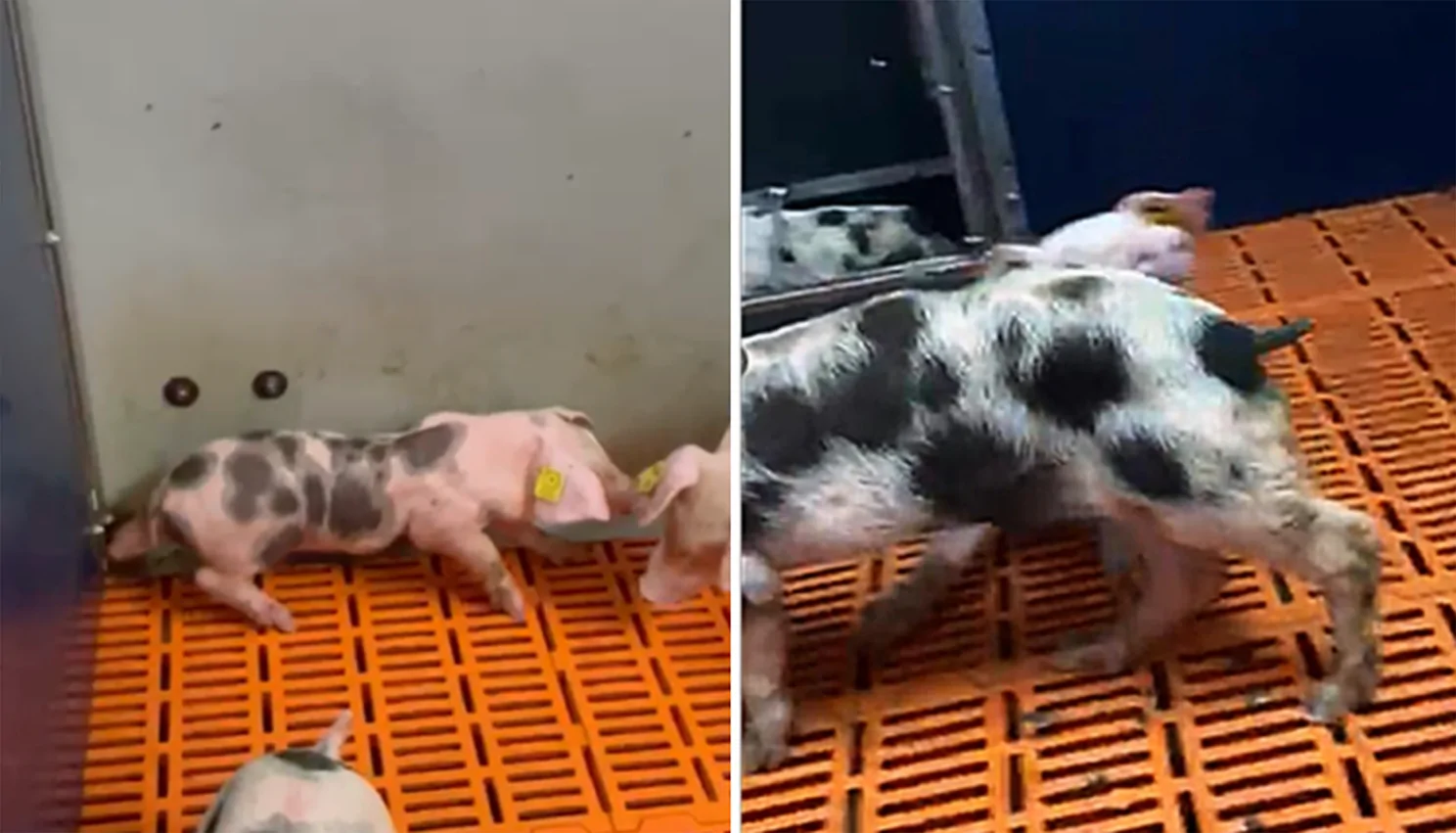
Recently weaned piglets with arched backs (left) and hind limbs tucked forward when walking (right)

After two weeks without improvement, the farm was re-visited by the responsible veterinarian and a close inspection of piglets’ behaviour at birth was made. Piglets were born apparently healthy and took colostrum on their first day of life with normality. However, after twenty-four hours, these same piglets showed apathy, ataxia, weakness and prostration, usually dying spontaneously on the third day of life. Clinical signs were not compatible with common nervous diseases in piglets, such as streptococcal meningitis or congenital tremors. No differences were found between the two sow genetic lines present in the farm or sow parity regarding piglet mortality. Besides, sows ate normally and were clinically unaffected. At the beginning of March, a closer examination of recently weaned piglets (thirty days of life) exhibited an arched back, reluctance to move, “goose stepping,” posterior weakness, and hind limbs tucked forward when walking (Fig. 2, Video).

Swine influenza virus (SIV), PRRSV and *Mycoplasma suis* infections were investigated by means of molecular biology (PCR or RT-PCR) methods in 10 affected, recently dead newborn piglets. Such analyses were performed on nasal swabs (SIV), lung (PRRSV) and serum (*M. suis* and PRRSV). Sow feed and drinking water from the farrowing crates were also sampled and tested for mycotoxins, microminerals and at a microbiological level.

### Post-mortem examination

Seven piglets from the farm were sent to the Veterinary Pathology Diagnostic Service (Faculty of Veterinary Medicine, *Universitat Autònoma de Barcelona*, Spain), where they were euthanised with an intravenous dose of pentobarbital through the cranial cava vein and subjected to a complete necropsy. Out of these, five displayed the previously mentioned clinical signs (three newborn and two recently weaned piglets) and two were apparently healthy (both newborns).

All necropsies performed on sick animals showed a slightly low body condition score (2 out of 5 [15]) and a generalized paleness of the skeletal muscle. One weaned piglet had an umbilical abscess and bilateral suppurative bronchopneumonia with fibrinous pleuritis, while the other one had a focal and small gastric ulcer. No other gross lesions were observed. Both piglets without clinical signs had a good body condition score (3 out of 5 [15]) and no relevant gross findings. The summary of the macroscopic and microscopic lesions can be found in Table 1.

The following tissue samples were collected and fixed by immersion in 10% buffered formalin for the microscopic study: mesenteric and superficial inguinal lymph nodes, lung, liver, spleen, heart, kidney, skeletal muscle (from forelimbs and hind limbs), nasal turbinates, stomach, ileum, brain, spinal cord (cervical, thoracic, and lumbar segments) and sciatic nerve. Tissues were trimmed, embedded in paraffin, cut into 4 μm sections, stained with haematoxylin and eosin, and studied under light microscope. Liver and spleen samples were taken for standard microbiologic culture to investigate an eventual septicaemic process.

**Fig. 3:**
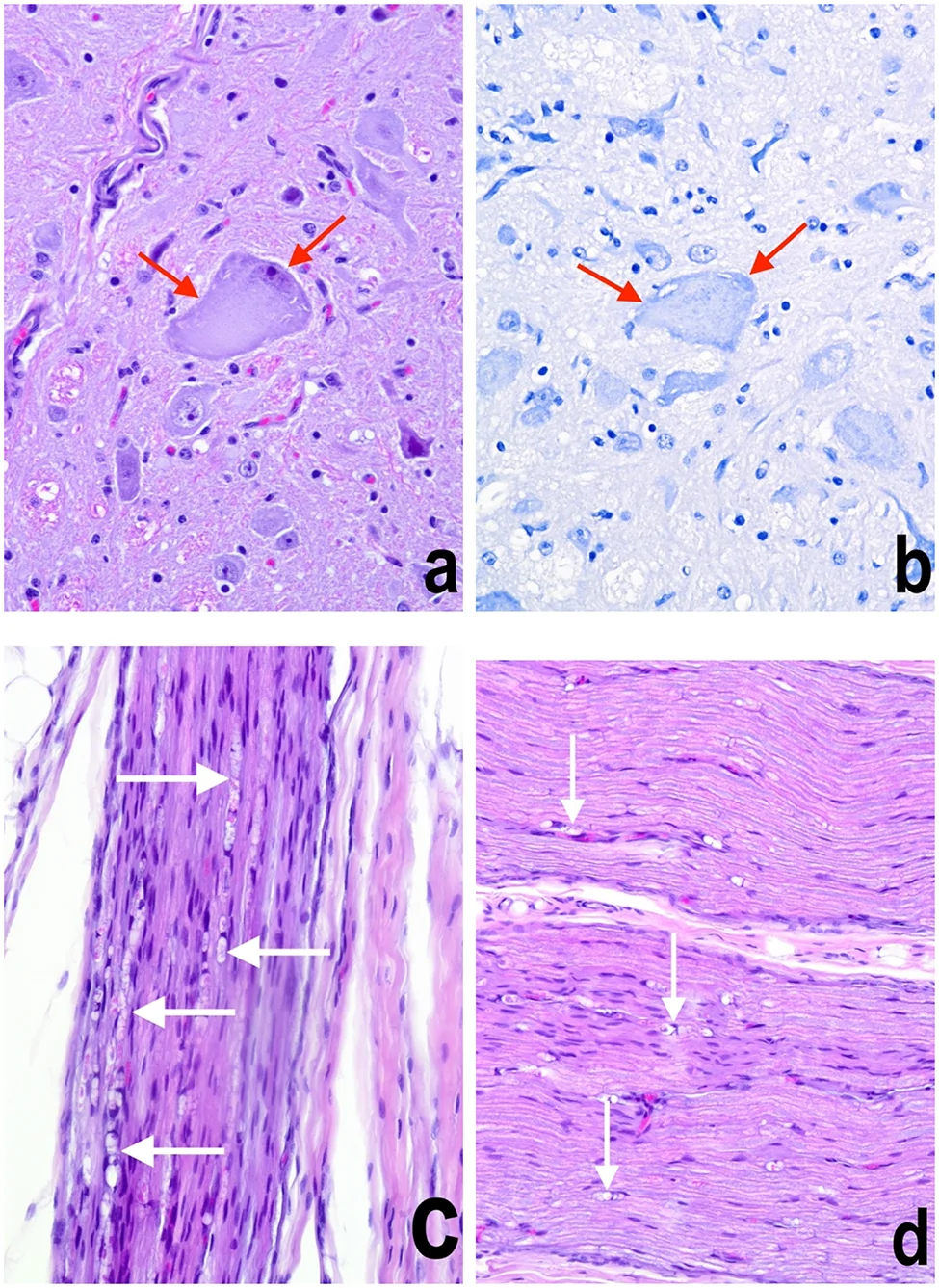
Microscopic lesions of the affected animals. **a**: Neuron from the ventral horn of cervical spinal cord showing central chromatolysis with the nucleus located at the periphery (red arrows). H&E stain. **b**: Nucleus located at the periphery of the cell body and absent and loss of Nissl granulation, from the ventral horn of cervical spinal cord (red arrows). Toluidine blue stain. **c**, **d**: Digestion chambers in sciatic nerves (white arrows). H&E stain

A variable proportion of neuronal somata of the grey matter of the spinal cord, both in the dorsal and ventral horns, showed pyknotic nuclei displaced to the periphery, and the cytoplasm was hyalinized with dissolution of the Nissl substance (central chromatolysis), as seen in Fig. 3a. Very few neurons also exhibited scarce clear microvacuoles in the cytoplasm or neuronal necrosis. Weaned piglets exhibited a more advanced picture of the lesion than the newborn ones. These findings tended to be moderate or severe in the cervical and thoracic segments and very subtle or absent in the lumbar ones (Table 1, Supplementary Table).

Additionally, representative sections of the spinal cord were stained with toluidine blue. Affected neurons were pale with nuclei located at the periphery of the cell body and absence or loss of Nissl granulation (Fig. 3b).

In the sciatic nerves of both weaned piglets, 20–30% of the axons were dilated, leaving empty spaces containing granular axon and myelin debris (Wallerian degeneration) often within the cytoplasm of macrophages (digestion chambers) (Fig. 3c and d). Newborn piglets had a milder or absent lesion. There were no lesions in skeletal muscle.

Healthy piglets had no microscopic lesions.

**Table 1:** Summary of the macroscopic and microscopic lesions found in each necropsied piglet during the post-mortem exam. Affected piglets were numbered from 1 to 5; piglets 6 and 7 were clinically normal

	Signalment	Macroscopic Lesions	Microscopic Lesions
Piglet 1	Female, weaned 4.36 kg	Suppurative bilateral bronchopneumonia with fibrinous pleuritis. Umbilical abscess.	Chromatolysis and neuronal necrosis of dorsal and ventral horn.Axonal degeneration of the sciatic nerve.Suppurative bilateral bronchopneumonia with fibrinous pleuritis.
Piglet 2	Male, weaned 4.10 kg	Gastric ulcer.	Chromatolysis and neuronal necrosis of the dorsal and ventral horn. Axonal degeneration of the sciatic nerve.
Piglet 3	Male, newborn 1.64 kg	None.	Neuronal necrosis of dorsal and ventral horn.
Piglet 4	Female, newborn 1.38 kg	None.	Neuronal necrosis of dorsal and ventral horn.
Piglet 5	Male, newborn 1.50 kg	None.	Chromatolysis and neuronal necrosis of the dorsal and ventral horn. Axonal degeneration of the sciatic nerve.
Piglet 6	Male, newborn 1.30 kg	None.	None.
Piglet 7	Female, newborn 1.92 kg	None.	None.

### Additional studies

SIV, PRRSV, or *M. suis* were not detected in any of the tested samples from affected piglets; liver and spleen samples taken in the postmortem exam for microbiologic culture to investigate an eventual septicaemic process were negative.

Since the only intake of newborns was sow milk, lactating mothers’ feed and drinking water were analysed in parallel to the post-mortem examinations of piglets.

A liquid chromatography-tandem mass spectrometry (LC MS-MS) of sow feed was carried out for mycotoxins and resulting values were below the maximum tolerated in each of them, too low to justify any clinical sign. Culture growth for *Escherichia coli* or *Clostridium perfringens* in sow’s feed was under maximum tolerance limits. Micromineral analysis by spectrophotometry on feed reported a deviation from the theoretical amount in the premix (excess of Cu x10, Fe x4, Mn x2 and Zn x2) probably due to the inclusion of a mineral supplement outside the formula. These values, however, were below potential toxicity levels.

Spectrophotometry of the drinking water revealed normal values for nitrates, nitrites, sulphates, ammonium, and chlorides. Only calcium seemed to be slightly above reference range (145 mg/L when the maximum recommended value is 100 mg/L) and pH was 5.9 (when normal range is 6.5–9.5). However, none of these results could be correlated to the clinical-pathological signs observed in the current case report. Microbiological quality, tested with standardculture and membrane filtration, was once again within normal values for total coliforms, *E. coli*, *C. perfringens* and its spores, and Enterococci.

Analysis of the sow feed confirmed that nutrient concentrations were within the recommended ranges described in the NRC Nutrient Requirements of Swine (2012) [16].

### Treatment and outcome

Since observed microscopic lesions were compatible with vitamin B5 deficiency [7, 9] and none of the findings regarding the lactating mother’s feed or drinking water could be associated with the clinical picture, parenteral vitamin B complex (Labidrosol B solution^®^ at 0.5 mL/piglet) was administered intramuscularly two to three times a week, starting by week 13 to all newborn piglets (first day of life). Each vial contained 20 mg/mL of vitamin B5; therefore, each piglet was injected with 10 mg per administration (20–30 mg per week). This intervention reduced first-week mortality from 41.2% (week 13) to 7.8% (week 14), as seen in Fig. 1. Three weeks later, in April, a temporary supply shortage of vitamin B complex led to clinical sign recurrence in three litters. In one of them, 100% of piglets died during their second day of life. The other two affected litters could be injected with vitamin B after twenty-four hours and all piglets recovered, reconfirming treatment effectiveness.

Based on the sharp effect of injected the vitamin B complex on piglet mortality, the assessment of calcium pantothenate concentration was carried out in the lactation diet of sows through a United States Pharmacopeia (USP) 39 method 91 analysis. This is a standardized microbiological assay used to quantify calcium pantothenate by measuring the growth response of a vitamin-dependent microorganism. Such analysis showed a value of 10.1 mg/kg of feed, despite the commercial premix theoretically being 12 mg/kg of feed. It was decided to increase the levels of PA in the ration up to 24.5 mg/kg, starting with the new lactation feed on the first week of July. The parenteral supplementation with vitamin B complex in piglets was stopped one month after introducing the new feed (first week of August) in lactation sows and no further cases appeared in newborn piglets.

## Discussion

This case report describes clinical, pathological and laboratory features of a vitamin B5 deficiency case in young piglets secondary to maternal dietary insufficiency, with sporadic occurrence observed in nursery pigs. Although PA deficiency has been previously reported [6, 7], this is, to our knowledge, the first description of its clinical progression over time (Fig. 1) and offers practical aspects of case management under field conditions.

The list of possible causes in neuro-locomotor conditions for pigs is considerably long [9]. Since the observed clinical-pathological picture in the herd was compatible with a metabolic or toxic process, the differential diagnosis included salt poisoning, also known as water deprivation or sodium ion intoxication, and micronutrient imbalances, such as deficiencies in calcium, copper, or vitamins B3, B5 or B6 [9]. Hepatic encephalopathy [9] was early ruled out due to the lack of significant lesions in the liver.

Newborns and already weaned animals displayed a “goose-stepping” walk [6, 9, 10], as well as weakness (see Video), and arched backs. This, alongside the histological observation of Nissl granules dissolution, necrosis of spinal cord neurons and Wallerian degeneration with digestion chambers in sciatic nerves, were strongly compatible of PA or vitamin B5 insufficiency [9]. All other pathological findings were considered incidental and not related to the neurological picture.

The clinical-pathological diagnosis was confirmed by the treatment efficacy, since there was an almost immediate decrease in neonatal mortality following intramuscular administration of vitamin B5 in these piglets. Moreover, the supply shortage that occurred, which triggered a temporary increase in mortality, also emphasized the attained diagnosis during weeks 18 and 19 (Fig. 1). Importantly, once the amount of PA in the sows’ feed was stabilized, neonatal mortality got back to normal levels without the need of PA parenteral injection in newborns.

Interestingly enough, B5 hypovitaminosis remained subclinical in sows, likely due to their minimal physical activity within the farrowing crates, which may reduce their metabolic need for PA. Meanwhile, neonatal piglets exhibited severe neurological affectations. This is explained by the role of vitamin B5 as a CoA precursor, since piglets require high rates of oxidative metabolism and energy production for thermoregulation and movement. Thus, the high metabolic turnover in these animals makes them more susceptible to the eventual development of neurological signs [9], as observed in the present case report.

According to the “New NRC (2012) Nutrient Requirements of Swine” [16], the total pantothenic acid required in pigs is between 7 and 12 mg/kg of diet. Piglets and lighter animals tend to need higher percentage of PA than older and heavier pigs [9, 16]. Most authors consider that NRC (2012) Nutrient Requirements of Swine can still be used for today’s hyperprolific sows, but only as a baseline framework, and not as a direct “plug‑and‑play” recommendation. In many situations, it will underestimate nutrient needs, especially in late gestation, nursery, and lactation for highly prolific modern genotypes [17, 18]. In the present case, considering that vitamin B5 levels in sows’ feed were lower than expected and the sharp effect of pantothenic acid administration in piglets, higher levels of such vitamin were implemented in sows’ ration. Moreover, the subsequent increased levels in their feed and the removal of vitamin B5 in piglets resulted in no further cases of the clinical condition.

Finally, although pandemic H1N1 influenza A virus infection occurred previously to the episode of PA deficiency, it was considered that no relationship between both conditions did exist.

## Conclusions

PA deficiency should be considered among the differential diagnoses of piglets with ataxia, muscle weakness and newborn mortality. A fast diagnosis based on complete nervous system histopathological assessment is key to diagnose the condition. The direct application of vitamin B5 in piglets was curative, while the increase of this vitamin in lactating of sows ensured counteracting the condition in the long term.

## Supplementary Information

Below is the link to the electronic supplementary material.


Supplementary Material 1



Supplementary Material 2


## Data Availability

All data generated in this work is included in the manuscript and its supplementary material.
