# Supplementary material for: A pantothenic acid deficiency outbreak causing mortality and neuromuscular disorders in newborn and nursery piglets
Source: Porcine Health Manag. 2026 Jun 17;12:47. doi: 10.1186/s40813-026-00527-z (PMC13528104; doi:10.1186/s40813-026-00527-z)
Supplement: Supplementary file 1 [file 40813_2026_527_MOESM2_ESM.docx]

**Supplementary Table**. Lesion intensity during microscopic study of the nervous system of each piglet. ++++ = severe; +++ = moderate; ++ = mild; + = discreet; − = no lesion.

|  | Signalment | Spinal cord | | | | | | Sciatic Nerve (sample from thigh) |
| --- | --- | --- | --- | --- | --- | --- | --- | --- |
|  |  | **Cervical** | | **Thoracic** | | **Lumbar** | |  |
|  |  | **Dorsal** | **Ventral** | **Dorsal** | **Ventral** | **Dorsal** | **Ventral** |  |
| Piglet 1 | Female, weaned  4.36 kg | ++ | ++++ | +++ | ++++ | + | + | +++ |
| Piglet 2 | Male, weaned  4.10 kg | ++ | ++ | + | + | - | - | ++ |
| Piglet 3 | Male, newborn  1.64 kg | ++ | ++ | + | ++ | ++ | ++ | - |
| Piglet 4 | Female, newborn  1.38 kg | ++ | ++ | +++ | +++ | ++ | ++ | - |
| Piglet 5 | Male, newborn  1.50 kg | - | - | +++ | +++ | ++ | ++ | ++ |
| Piglet 6 | Male, newborn  1.30 kg | - | - | - | - | - | - | - |
| Piglet 7 | Female, newborn  1.92 kg | - | - | - | - | - | - | - |
